# Impact of 6-Hour Sepsis Resuscitation Bundle Compliance on Hospital Mortality in a Saudi Hospital

**DOI:** 10.1155/2012/273268

**Published:** 2012-10-03

**Authors:** Javed I. Memon, Rifat S. Rehmani, Abdulsalam M. Alaithan, Ayman El Gammal, Talib M. Lone, Khaled Ghorab, Abdulsaboor Abdulbasir

**Affiliations:** ^1^Intensive Care, Department of Medicine, King Abdulaziz Hospital, P.O. Box 2477, Al-Ahsa, Saudi Arabia; ^2^Department of Emergency Medicine, King Abdulaziz Hospital, Al-Ahsa, Saudi Arabia; ^3^Infectious Diseases/Department of Medicine, King Abdulaziz Hospital, Al-Ahsa, Saudi Arabia

## Abstract

*Purpose*. To assess the effect of improved compliance with 6-hour sepsis resuscitation bundle on mortality in patients with severe sepsis and septic shock. *Materials and Methods*. A quasi-experimental prospective study was conducted at a 10-bedded combined medical and surgical intensive care unit. The historical group included all consecutive patients with severe sepsis and septic shock admitted from January 2008 to March 2009. Intervention included evidence-based written sepsis pathway, antibiotic recommendations, and an educational program.The post-intervention group included all consecutive patients admitted from July 2009 to June 2011. The primary outcome measures were the overall compliance to seven 6-hour sepsis resuscitation bundle elements and 30-day hospital mortality. There were 99 patients in the historical group and 199 in the post-intervention group. *Results*. The baseline patients' characteristics were similar. Overall compliance to all seven sepsis resuscitation bundle elements in historical group was 5.1% [95% confidence interval (CI), 2.1–11.3] which improved after intervention to 23.6% (95% CI, 17.9–30.1); *P* < 0.001. The overall compliance to 6-hour sepsis resuscitation bundle elements was associated with improved survival [odds ratio (OR), 5.8 (95% CI, 2.2–15.1; *P* < 0.001)]. 30-day hospital mortality reduced from 31.3% in the historical group to 21.1% in the intervention group; *P* = 0.05. *Conclusion*. Improvement in compliance to 6-hour sepsis resuscitation bundle was associated with a reduction in 30-day hospital mortality.

## 1. Introduction

Over the last decade the incidence of sepsis has increased, with higher hospitalization rates and increased disease severity [1–4]. One in every four to five admissions to intensive care units (ICUs) is related to severe sepsis and septic shock [1, 3, 5]. The associated mortality rates due to severe sepsis and septic shock varies from 25 to 70% [6, 7]. Surviving Sepsis Campaign (SSC) was launched in 2002, aiming to accomplish a 25% reduction in the relative mortality in sepsis during a five year period by means of improvement in the recognition and treatment of sepsis [8]. The SSC initially published evidence-based guidelines in 2004 and were updated in 2008 to improve the standardized care in the form of sepsis bundles to improve outcomes in severe sepsis and septic shock [9]. A bundle is a group of interventions related to a disease process and when executed together produce better outcomes than when implemented individually. The sepsis-bundled care has not only shown a reduction in mortality, [10–14] but is also cost-effective in developed countries [15, 16]. The compliance to these guidelines and the outcome to compliance has been reported from a developing country like Brazil [17], and only one multicenter study has reported the compliance to sepsis bundle from Asian ICUs, including ICUs from the Saudi Arabia [18]. We conducted a quasi-experimental study to assess the effectiveness of 6-hour sepsis resuscitation bundle with regard to both implementation and outcome in terms of mortality reduction in the management of patients with severe sepsis and septic shock.

## 2. Materials and Methods

### 2.1. Design and Setting

This was a quasi-experimental prospective study that included a post-intervention group and a historical group. The study was conducted at a 10-bedded combined medical and surgical ICU in King Abdulaziz Hospital Al-Ahsa, Saudi Arabia. 

The historical group included all consecutive patients with severe sepsis and septic shock admitted to the ICU over a 15-month period before the implementation of the sepsis pathway (January 2008–March 2009). The intervention was introduced over a 3-month period during which no patient data were collected. The post-intervention data was collected from all consecutive patients with severe sepsis and septic shock admitted to the ICU over a 24-month period (July 2009 to June 2011) after the implementation of the sepsis pathway. 

#### 2.1.1. Definitions

The definitions of sepsis, severe sepsis, and septic shock were adapted mostly from international sepsis definitions conference and the Surviving Sepsis Campaign [9, 19, 20]. Sepsis was defined as suspected infection with two or more out of the four systemic inflammatory response syndrome (SIRS) criteria, that is: temperature >38 or <36°C, heart rate > 90 beats per minute, respiration >20 breaths per minute and white blood cell counts >12,000 or <4000/mm^3^ or >10% band forms. Severe sepsis was defined as sepsis plus one or more organ dysfunction variables including; Hypotension [systolic blood pressure (SBP) <90 mmHg or mean arterial pressure (MAP) <65 mmHg or SBP decrease >40 mmHg from baseline], acute alteration in mental status; acute lung injury with ratio of the partial pressure of arterial oxygen to fractional inspired oxygen ≤300; creatinine >2.0 mg/dL (176.8 mmol/L) or creatinine increase of 0.5 mg/dL (45 mmol/L) from baseline or urine output <0.5 mL/kg/hour for >2 hours; international normalized ratio >1.5 or partial thromboplastin time > 60 seconds; acute reduction of platelet count <100,000/*μ*L; total bilirubin >35 mmol/L and lactate > 2 mmol/L (18 mg/dL). Septic shock was defined as sepsis-induced persistent hypotension despite adequate fluid resuscitation.

#### 2.1.2. 6-Hour Sepsis Resuscitation Bundle

The seven elements of the resuscitation bundle described by SSC are serum lactate measured; blood cultures obtained prior to antibiotic administration; broad-spectrum antibiotics administered within 3 hours for emergency department (ED) admissions and 1 hour for non-ED admissions; in the event of hypotension and/or lactate ≥4 mmol/L, deliver an initial minimum of 20 mL/kg of crystalloid, initiate vasopressor for hypotension not responding to initial fluid resuscitation to maintain MAP ≥65 mmHg; in the event of persistent hypotension despite fluid resuscitation (septic shock) and/or lactate >4 mmol/L, achieve central venous pressure (CVP) of ≥8 mmHg and achieve central venous oxygen saturation ScvO2 ≥70% [20]. 

### 2.2. Sample Size

Our baseline mortality for severe sepsis and septic shock is 30%. The sample size was calculated by the PASS program, based on the hypothesis that the implementation of the protocol would reduce the mortality by 25% (i.e., a decrease from 30% at baseline to 22% after intervention). This implied a sample size of 193 subjects given 80% power and type 1 error rate of 0.05.

### 2.3. Intervention

A special task force team was put together in April 2009 to prepare the evidence-based sepsis pathway for the management of patients with severe sepsis or septic shock. The task force team developed a written protocol based on the most recent guidelines [9], as well as intravenous antibiotic recommendations based on presumed source of infection. An educational program in the form of lectures was also designed for the physicians, nursing and respiratory colleagues. The process continued until the end of June 2009 and the written protocol was implemented in the ICU, ED and general medical wards from July 1, 2009. 

### 2.4. Eligibility

We screened all patients ≥18 years who presented with (a) suspected infection, (b) ≥2 SIRS criteria, (c) ≥1 organ dysfunction, (d) lactate level >4 mmol/L, or (e) hypotension for eligibility. Exclusion criteria included acute cardiogenic pulmonary edema, acute coronary syndrome, acute cerebral vascular event, pregnancy, drug overdose, burn injury, trauma, requirement of immediate surgery, and “do not resuscitate” (DNR) status at presentation with sepsis or DNR within 6 hours of presentation with sepsis. If a patient had more than 1 episode of sepsis within the same admission, only the data from the initial episode was used for the final analysis.

The study was carried out according to the principles of the Helsinki Declaration, and was approved by the Institutional Research Committee.

### 2.5. Data Collection

All ICU admissions during the study period were actively screened for the presence of severe sepsis or septic shock using a screening tool. One of the co-investigators made daily rounds to all study patients to abstract relevant data from the medical records and the bedside flow sheets. Demographical data of the historical group were obtained from the ICU database and review of the medical records was carried out to collect the timelines of the various interventions from time zero.

The clinical and demographic characteristics of all patients, including age, sex, sepsis screening parameters, acute physiology and chronic health evaluation II (APACHE II) score for assessment of severity of illness, admission source, comorbidities (diabetes mellitus, hypertension, chronic kidney disease, and malignancy) and source of infection at sepsis presentation were recorded. The need for vasopressor agents and vasopressor days, necessity for continuous renal replacement therapy (CRRT) or mechanical ventilation (MV) during the ICU stay, and days of MV was also recorded. All data were entered into a dedicated computerized database (Microsoft Access, Microsoft Corporation, Redmond, WA, USA).

The time zero was defined as the time when severe sepsis or septic shock was recognized and sepsis resuscitation bundle initiated. If no time and date could be found by searching the chart, the default time of presentation was the time of admission to the ICU.

### 2.6. Outcome Measures

The compliance to the 6-hour sepsis resuscitation bundle was measured at two levels. Firstly, we measured the compliance of seven individual bundle elements, and secondly, we also measured the overall compliance of the resuscitation bundle (compliance with all seven elements of the 6-hour sepsis resuscitation bundle).

The primary outcome measures were overall compliance to 6-hour resuscitation bundle elements and 30-day hospital mortality. For each element of the resuscitation bundle, the patient was scored 1 for compliance and was scored 0 for non-compliance. All elements were to be completed along with predetermined hemodynamic targets within 6 hour of activation of sepsis pathway. Secondary outcome measures included ICU mortality, ICU and hospital length of stay.

### 2.7. Statistical Analysis

Statistical analysis was done with the Statistical Package for the Social Sciences (SPSS), Version 18.0. Continuous data are described by mean and standard deviation, and categorical data as numbers (percentage and 95% confidence interval for compliance with bundle elements); baseline characteristics and outcomes of patients in the two time periods were compared using the chi-squared or Fisher's exact tests for categorical variables and the *t*-test for continuous variables. Values of *P* < .05 were considered significant. 

Multivariable logistic regression modeling was used to identify the independent relationship between overall compliance with all bundle elements as an independent variable and the mortality in the study cohort. Variables assessed as confounders were those that were significantly different in the pre- and post-intervention groups. A *P* value of0.1 was selected as the criterion for retention of variables in the model to ensure that potential confounders were not excluded.

## 3. Results

**Figure 1:**
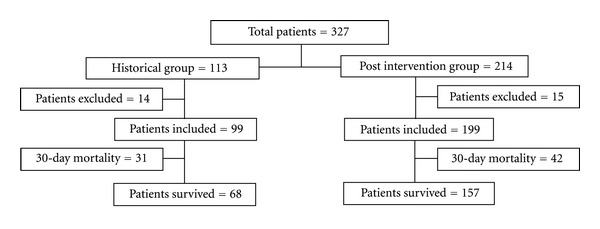
Distribution of patients evaluated by study groups.

The distribution of patients in historical and post-intervention groups is shown in Figure 1. The baseline patients' characteristics in the historical as well as in the post-intervention group were similar (Table 1), with no statistically significant differences in age, sex, admission source, screening parameters, septic shock, or APACHE II score. The main sources of sepsis were pneumonia and urinary tract infections in both periods. Pneumonia was more frequent in the post-intervention cohort but the difference was statistically nonsignificant. There were no significant differences in comorbidities at sepsis presentation. 

Mechanical ventilation (MV) was required in 54 (55%) of patients in the historical group compared to 90 (45%) in the post-intervention group which was statistically nonsignificant, however, mean days of MV were 5.8 ± 9.5 in the historical group compared to 3.4 ± 6.7 in the post-intervention group, with a *P *value* of* 0.01. Similarly, mean vasopressor days in the historical group were 4.4 ± 6.2 compared to 2.9 ± 5.1 in the post-intervention group; *P* = 0.03. The use of CRRT was comparable in the two groups. 

The compliance to the individual elements of the resuscitation bundle in both groups is presented in Table 2.

At baseline, overall compliance to resuscitation bundle was only 5.1% [95% confidence interval (CI), 2.1–11.3] which improved significantly to 23.6% (95% CI, 17.9–30.1) after the intervention. Also, the overall compliance to resuscitation bundle was related to improved survival [odds ratio (OR), 5.8; 95% CI, 2.2–15.1; *P* < 0.001].

We found low compliance in the historical cohort with administration of appropriate broad spectrum antibiotics at allocated times and achieving targeted CVP and ScvO2. The compliance to all the elements of resuscitation bundle improved after the intervention especially, the administration of appropriate broad spectrum antibiotics and ScvO2.

We also found that the survival was related to the completion of an increasing number of bundle element compliance, as shown in the Table 3.

The following potentially confounding variables identified were evaluated in the logistic regression analysis: congestive heart failure, respiratory failure, and increasing compliance with the bundle elements (1–4 versus 5–7). In addition, CRRT was included as a possible confounder because it may be an independent predictor of adverse events. The following covariates, in order of importance, were retained in the model: CRRT (*P* = 0.001) and increasing compliance with the bundle elements (1–4 versus 5–7) (*P* = 0.017). Overall compliance with the bundles also remained in the model with a *P* value of 0.02 and OR for the composite adverse event of 0.41 (95% Cl,0.17–0.84).

There was a significant 30-day hospital mortality reduction in the post-intervention group as shown in Table 4. The secondary outcome measures, including ICU mortality, ICU and hospital stay were not significant statistically as shown in Table 4.

## 4. Discussion

Our study showed that implementation of the sepsis pathway in the general hospital improved the compliance to 6-hour sepsis resuscitation bundle according to SSC guidelines which lowered the 30-day hospital mortality. The compliance rate improved five times after the implementation of the sepsis pathway and the reduction in 30-day hospital mortality was statistically significant. The improvement in the compliance rate to lactate measurement, broad spectrum antibiotics administration in the allocated time, intravenous fluid delivery, and achieving targeted ScvO2 after the intervention was statistically significant. The improved overall compliance to the resuscitation bundle was related to six times improved survival. Moreover, there was consistent increase in survival with the increasing compliance to the number of elements of 6-hour resuscitation bundle. The possible explanation is more awareness of the disease management has led to more therapeutic interventions in a timely manner, which was reflected in an improvement in the overall survival.

Prior to the implementation of written sepsis pathway and educational program, our overall compliance was not good. This has been addressed in the literature; compliance to resuscitation bundle from Asian ICUs was reported to be 7.6% only [18]. A multicenter study from Spain also revealed a low baseline adherence to sepsis resuscitation bundle of 5.3% [21]. A recent survey conducted in the emergency departments of the 25 most densely populated areas in the United States to identify barriers to implementation of a written protocol for early goal-directed therapy (EGDT) for severe sepsis revealed that 23% of the surveyed hospitals were not using or even planning on developing a protocol for EGDT in sepsis [22]. Another emergency medicine survey including physicians from 30 academic tertiary care hospitals revealed that only 7% use EGDT [23]. Despite overall low compliance to all the elements of the resuscitation bundle, basline 30-day mortality in our hospital was not very high. Possible reason being that the emergency medicine and critical care physicians were targeting early perfusion despite the fact that there was no written resuscitation or management protocol.

The improved compliance with the resuscitation bundle after our intervention led to a 30-day hospital mortality reduction. Castellanos-Ortega and his colleagues reported an increase in 6-hour resuscitation bundle compliance from 1% to 11%, leading to significant mortality reduction [13]. Nguyen et al. showed a significant reduction in mortality in severe sepsis and septic shock patients with the bundle completed at 6 hours (20.8%) compared to the bundle not completed (39.5%) [10]. A prospective study from the United Kingdom reported that in patients who completed 6-hour bundle, there was reduction in mortality from 49% to 23%, however, their bundle was different from the sepsis resuscitation bundle as they used a hemoglobin target of 7 to 9 g/dL instead of a hematocrit of >30% and used the remaining hypotension after the fluid resuscitation for the threshold of inotropes instead of ScvO2 [11]. A study from Switzerland showed lower in-hospital mortality in patients who completed all 6 hour bundle elements compared to those who missed one or more elements, that is, 11.4% versus 31.3% [24]. The reasons of mortality reduction in our study were mainly due to improvement of fluid delivery, timely infusion of appropriate antibiotics, and achievement of targeted ScvO2. Kumar et al. showed that each hour of delay in antibiotic administration in septic shock patients at the onset of hypotension was associated with an 8% decrease in survival rate [25]. A study from Spain showed that the greatest benefit among the elements of 6-hour resuscitation bundle was achieved by accomplishing the target ScvO2 of ≥70% [13].

Our study shows an improvement in survival with increasing compliance to the number of elements of 6-hour resuscitation bundle. The study from Spain showed that the completion of more than any four elements of 6-hour resuscitation bundle was associated with reduced mortality, with a highest probability of survival in patients who completed six or more interventions [13]. The retrospective study of surgical septic shock patients also revealed that the survival was significantly related to the achievement of an increased number of therapeutic criteria which included both the 6-hour resuscitation and 24-hour management bundle [12].

Our study did not reveal any significant differences in the secondary outcomes, including ICU mortality, ICU and hospital length of stay. Considering significant reduction in the mechanical ventilation and vasopressor days in the intervention group, one can assume that ICU and hospital length of stay should have been reduced as well. Similar studies on sepsis bundles did not find any influence on hospital length of stay either [10, 26]. One possible explanation could be the nonavailability of high dependency beds, delaying transfers from the ICU, as our hospital bed occupancy rate is very high. Also, we do not have nursing homes regionally, therefore patients cannot be discharged in a timely fashion from the wards if they cannot be properly looked after at home, leading to prolonged hospital stays. 

Our study has some limitations that need to be considered. Missing information in the medical records could have affected the compliance rate and mortality in the historical arm of the study. We did not categorize whether the infection was hospital- or community-acquired, which might have affected the outcome in terms of mortality. Also, we did not include the compliance to 24-hour management bundle in our study as this was not our study objective and 6-hour resuscitation bundle has shown more effectiveness compared to 24-hour management bundle [13]. Finally, this is a single-center study, and therefore the results may not be generalized to the entire Kingdom of Saudi Arabia.

## 5. Conclusion

The improvement in overall compliance with 6-hour sepsis resuscitation bundle significantly reduced the 30-day hospital mortality in patients with severe sepsis and septic shock. 

## Figures and Tables

**Table 1:** Patients characteristics and clinical data.

Variables	Historical group (*n* = 99)	Post-intervention group (*n* = 199)	*P* value
Age	68.6 ± 18.3	65.0 ± 20	0.13
Sex (male)	50 (51)	110 (55)	0.46
Septic shock	62 (63)	144 (72)	0.11
Sepsis screening			
Mean arterial pressure (mmHg)	59.6 ± 15.6	56.7 ± 11.0	0.06
Heart rate (beats/min)	110 ± 24.0	106 ± 24.0	0.07
Respiratory rate (breaths/min)	26.0 ± 7.6	26.1 ± 7.7	0.57
Temperature (°C)	37.1 ± 1.2	37.1 ± 1.0	0.49
White-cell count (per mm^3^)	15.6 ± 10.1	14.5 ± 8.6	0.32
Severity of illness			
APACHE II score	21.6 ± 7.4	21.2 ± 7.0	0.22
Admission source			
Emergency department	46 (46.4)	108 (54.2)	0.08
Wards	53 (53.5)	91 (45.7)	0.08
Co-morbid conditions			
Diabetes mellitus	62 (62)	124 (62)	0.88
Hypertension	63 (63)	128 (64)	0.79
Chronic kidney disease	16 (16)	28 (14)	0.48
Malignancy	12 (12)	22 (11)	0.34
Source of infection			
Urinary tract	21 (21)	36 (18)	0.36
Pneumonia	27 (28)	67 (34)	0.14
Abdomen	19 (19)	24 (12)	0.12
Soft tissue and skin	4 (4)	21 (11)	0.03
Others, with undetermined source	11 (11)	18 (9)	0.64
Mixed (>one source)	17 (17)	33 (16)	0.79
Other variables			
Lactate (mmol/L)	3.9	3.5	0.09
Mechanical ventilation	54 (55)	90 (45)	0.11
Need for vasopressors	64 (67)	153 (76)	0.09
Mechanical ventilation days	5.8 ± 9.5	3.4 ± 6.7	0.01
Vasopressor days	4.4 ± 6.2	2.9 ± 5.1	0.03
CRRT	12 (12)	24 (12)	0.94

Results are expressed as Mean ± SD or *n* (%), APACHE: acute physiology and chronic health evaluation, CRRT: continuous renal replacement therapy.

**Table 2:** Compliance with 6-hour sepsis resuscitation bundle elements.

Bundle elements	Historical *n* (%)	Post-intervention *n* (%)	*P* value
Serum lactate measured	83 (83.8)	197 (99.0)	0.001
Blood cultures before antibiotics	83 (83.8)	173 (87.0)	0.47
Appropriate broad-spectrum antibiotics administration in allocated time	41 (41.4)	153 (76.6)	0.005
Intravenous fluids delivered	81 (81.8)	183 (92.0)	0.009
Mean arterial pressure ≥65 mmHg achieved	88 (88.8)	185 (94.0)	0.27
Central venous pressure ≥8 mmHg achieved	44 (53.1)	87 (55.7)	0.9
Central venous oxygen saturation ≥70% achieved	10 (10.1)	104 (50.3)	0.003
Overall compliance (all 7 bundle elements completed)	5 (5.1)	47 (23.6)	0.001

*n*: number.

**Table 3:** Association between the increasing number of resuscitation bundle elements compliance and the survival in both groups.

Compliance with number of elements of resuscitation bundle	Survival *n* (%)	95% CI
1 (*n* = 1)	0 (0)	0-0
2 (*n* = 4)	1 (25.0)	34.1–71.0
3 (*n* = 27)	15 (55.5)	37.3–72.4
4 (*n* = 66)	45 (67.4)	56.2–78.2
5 (*n* = 90)	72 (80.0)	70.5–87.0
6 (*n* = 58)	45 (78.0)	65.2–86.5
7 (*n* = 52)	44 (84.6)	72.2–92.3

*n*: number of patients, CI: confidence interval.

**Table 4:** Outcome measures.

Outcome variables	Results		*P* value
	Historical group	Post-intervention group	
Primary outcome			
30-day hospital mortality	31/99 (31.3%)	42/199 (21.1%)	0.05
Secondary outcomes			
ICU mortality	27 (27.3%)	39 (19.6%)	0.11
Hospital stay, days	21.1 ± 19.6	21.8 ± 19.4	0.89
ICU stay, days	8.2 ± 7.9	7.6 ± 8.3	0.53

Mortalities are described as *n* (%); Stays are described as mean ± SD, ICU: intensive care unit.
